# An Advance Movement

**Published:** 1901-03

**Authors:** 


### Editorial.

### AN ADVANCE MOVEMENT.

It will be observed by the title-page of this month's Journal that the International has taken a new step forward in its work by associating with it several collaborators to prepare useful and interesting matter for its pages.

A new department is organized, to be devoted to reviews of important papers appearing in the dental journals here and abroad. These reviews are expected to present the advancement of dentistry to the readers of the International in order to elucidate the views of the leading writers in a synoptical manner without carrying over their language,—to be, in other words, such a presentation as is made in a bibliographical review, by stating in a compendious way the salient features of the subject under treatment. After the analysis is made the application of general principles may be given to exhibit the value of the article.

The intention is to include in these reviews not only the valuable papers in the English language, but those in the German and the French. The matter for this department will be prepared by Dr. Briggs and Dr. Potter, who are by education and training well qualified. The effect of this effort will be to give the JOURNAL more fully an international character by making its pages the repository of the better views of the world upon dental subjects.

To Dr. McClain will be assigned the preparation of miscellanea. His endeavors will be to seek useful information from various sources, principally from dental literature, as may be serviceable, more particularly to that large class of our profession whose habits of work are forming and who look for practical methods to enlighten the difficulties that arise in practice.

The underlying end in the production of the INTERNATIONAL is that the dental profession may have a journal conducted under its own auspices, but this craving to satisfy a feeling of self-respect would be of little value unless at the same time every effort were made to furnish not only most ably written papers bearing on dental science, but those of a helpful character. The editor and the managers of the JOURNAL will endeavor to induce the preparation for its pages of short articles upon practical subjects. We appeal to the society members this magazine represents, and its readers, to contribute this character of matter for publication by us.

The INTERNATIONAL has become an important factor in dental affairs, and appeals to all interested in dental progress to assist in the labor which should be considered a duty common to us all,—managers, society members, readers, and the profession generally.

INTERNATIONAL DENTAL PUBLISHING COMPANY.

